# Accurate molecular classification of cancer using simple rules

**DOI:** 10.1186/1755-8794-2-64

**Published:** 2009-10-30

**Authors:** Xiaosheng Wang, Osamu Gotoh

**Affiliations:** 1 Department of Intelligence Science and Technology, Graduate School of Informatics, Kyoto University, Kyoto 606-8501, Japan; 2 National Institute of Advanced Industrial Science and Technology, Computational Biology Research Center, Tokyo 135-0064, Japan

## Abstract

**Background:**

One intractable problem with using microarray data analysis for cancer classification is how to reduce the extremely high-dimensionality gene feature data to remove the effects of noise. Feature selection is often used to address this problem by selecting informative genes from among thousands or tens of thousands of genes. However, most of the existing methods of microarray-based cancer classification utilize too many genes to achieve accurate classification, which often hampers the interpretability of the models. For a better understanding of the classification results, it is desirable to develop simpler rule-based models with as few marker genes as possible.

**Methods:**

We screened a small number of informative single genes and gene pairs on the basis of their depended degrees proposed in rough sets. Applying the decision rules induced by the selected genes or gene pairs, we constructed cancer classifiers. We tested the efficacy of the classifiers by leave-one-out cross-validation (LOOCV) of training sets and classification of independent test sets.

**Results:**

We applied our methods to five cancerous gene expression datasets: leukemia (acute lymphoblastic leukemia [ALL] vs. acute myeloid leukemia [AML]), lung cancer, prostate cancer, breast cancer, and leukemia (ALL vs. mixed-lineage leukemia [MLL] vs. AML). Accurate classification outcomes were obtained by utilizing just one or two genes. Some genes that correlated closely with the pathogenesis of relevant cancers were identified. In terms of both classification performance and algorithm simplicity, our approach outperformed or at least matched existing methods.

**Conclusion:**

In cancerous gene expression datasets, a small number of genes, even one or two if selected correctly, is capable of achieving an ideal cancer classification effect. This finding also means that very simple rules may perform well for cancerous class prediction.

## Background

Rapid advances in gene expression microarray technology have enabled the simultaneous measurement of the expression levels of tens of thousands of genes in a single experiment [1]. By measuring gene expression levels related to multiple individuals and multiple tissue or tumor samples, investigators can discover molecular markers to be used for cancer diagnosis, prognosis, and prediction. Many researchers have explored the use of microarray technology to build cancer diagnosis, prognosis, and prediction classifiers, since the pioneering work of Golub et al. in applying gene expression monitoring by DNA microarray to cancer classification [2]. However, one intractable problem with using microarray data analysis to create cancer classifiers is how to reduce the exceedingly high-dimensional gene expression data, which contain a large amount of noise. On the other hand, compared with the measured quantities of gene expression levels in experiments, the numbers of samples are severely limited. This brings about two computational challenges: computational cost and classification accuracy. To achieve efficient and accurate classification, it is natural for researchers to investigate feature selection; i.e., gene filtering [3]. However, one serious drawback of most existing methods is that too many genes are ultimately selected for the classification of cancer, thereby hampering the interpretability of the models. In fact, it is not easy to gauge which gene is essential in determining a cancerous class if accurate classification is obtained based on a large cluster of genes.

In parallel with feature selection, classifier construction is an important topic in this field. In machine learning and data mining, the methods of generating classifiers include unsupervised and supervised approaches. The latter is further classified into two categories: "black-box" and "white-box" models. The "black-box" models, such as support vector machines (SVMs), discriminant analysis (DA), artificial neural networks (ANNs), genetic algorithms (GAs), naïve Bayes (NB), and *k*-nearest neighbors (*k*-NNs), address classification problems without any knowledge-based explanation rules. In contrast, the "white-box" models, such as Decision Trees [4], Rough Sets [5], and emerging patterns (EPs) [6], often implement classification by giving "IF-THEN"-like rules. The "white-box" models are sometimes more welcomed by biologists and clinicians because they are easily understood.

Many investigators have utilized the rule-based approaches (i.e., "white-box" models) to produce cancer classifiers [6-13]. In general, these classifiers involve few genes, whereas they exhibit efficient prediction performance. In [6], the authors proposed one method of identifying good diagnostic gene groups from gene expression profiles using the concept of EPs. The authors sought to find the gene groups whose frequency of patterns changed significantly between two classes of cells. They then used the rules arising from these patterns to construct cancer classifiers. Their classifiers were uncomplicated, as they merely contained the rules involving a few genes. In [11], decision tree algorithms involving single C4.5, Bagging, and AdaBoost decision trees were applied to classify gene expression datasets. In essence, a decision tree is a rule-based classifier. The classifier screens the informative features to build decision trees based on the information entropy concept. Subsequently, rules are derived from the trees. Because decision tree algorithms commonly conduct pruning of the trees to remove unnecessary features, the derived rules generally involve only a small number of features. In [13], the authors proposed the use of high-ranked association rule groups to construct cancer classifiers instead of utilizing all of the mined association rules, which commonly involves excessive numbers of redundant rules.

Some investigators have addressed the problem of using pairs of genes to conduct cancer classification. In [14], the authors classified gene expression profiles using a comparison-based approach, the "top-scoring pair(s)," called the *TSP *classifier. The authors attempted to predict classes by comparing the expression levels of a single pair of genes, chosen based on a simple measure of class discrimination. In [15], the authors investigated the use of gene pairs for classification. They screened the gene pairs that had marked differences in average expression levels between the tumor types in the training set. The gene pairs were then applied to classify test sets.

Rough sets, a data-analysis method originally proposed by Pawlak in the early 1980s [5], has evolved into a widely accepted machine-learning and data-mining method [16]. In [7-10], rough sets was applied for cancer classification and prediction based on an attribute reduction approach. In [17], we proposed a rough sets-based soft computing method to conduct cancer classification using single genes or gene pairs. In this article, we also explore the use of single genes and gene pairs in constructing cancer classifiers; however, in contrast to [17], we first aimed to use the concept of canonical depended degree, as proposed in rough sets for gene selection. In the cases that this approach was unsuccessful, we considered utilizing the *α *depended degree standard suggested in [17] for gene selection. In this work, the *α *depended degree was employed for a portion of the datasets. In addition, unlike the other rough sets-based methods, we did not carry out attribute reduction for gene selection. Instead, we first implemented feature ranking according to the depended degree or *α *depended degree of attributes, and then selected the top-ranked genes to create classifiers so as to avoid expensive computation for attribute reduction. Moreover, we made use of the decision rules induced by the chosen genes to build classifiers, whereas existing rough sets-based methods only utilized rough sets for gene selection, and the classifier constructions depended upon other machine-learning algorithms such as SVMs, ANNs, GAs, NB, and *k-*NNs [7-10].

We tested the methods in the five publicly available gene expression datasets: Leukemia 1 (ALL vs. AML), Lung Cancer, Prostate Cancer, Breast Cancer, and Leukemia 2 (ALL vs. MLL vs. AML), which can be downloaded from the Kent Ridge Bio-medical Data Set Repository . We compared our results with the findings of previous studies. Furthermore, we examined and analyzed the biological relevance of the selected genes.

## Methods

### Rough sets

In rough sets, an equivalence relation on *U *is referred to as one *knowledge*, and a family of equivalence relations is referred to as a *knowledge base *on *U*. In reality, we are often faced with a large amount of ill-defined data, and we want to learn about them based on pre-existing knowledge. However, most of these data cannot be precisely defined based on pre-existing knowledge, as they incorporate both definite and vague components. In [5], Pawlak describes the definite parts using the concept of positive region.

**Definition 1 **Let *U *be a universe of discourse, *X *⊆ *U*, and *R *is an equivalence relation on *U*. *U/R *represents the set of the equivalence class of *U *induced by *R*. The *positive region *of *X *on *R *in *U *is defined as *pos(R, X) *= ∪ {*Y *∈ *U/R *| *Y *⊆ *X*} [5].

The decision table is the data form studied by rough sets. One decision table can be represented as *S *= (*U*, *A *= *C *∪ *D*), where *U *is the set of samples, *C *is the condition attribute set, and *D *is the decision attribute set. Without loss of generality, hereafter we assume *D *is a single-element set, and we call *D *the *decision attribute*. *A *can be viewed as a knowledge base in *S*, as each attribute or attribute subset can induce an equivalence relation on *U*. In the decision table, if we designate *I*_*a *_as the function mapping a member (sample) of *U *to the value of the member on the attribute *a *(*a *∈ *A*), then the equivalence relation *R(A') *induced by the attribute subset *A' *⊆ *A *is defined as: for ∀*x*, *y *∈ *U*, *xR(A')y*, if and only if *I*_*a*_*(x) *= *I*_*a*_*(y) *for each *a *∈ *A'*.

For the cancer classification problem, every collected set of microarray data can be represented as a decision table in the form of Table 1. In the microarray data decision table, there are *m *samples and *n *genes. Every sample is assigned to one class label. The expression level of gene *y *in sample *x *is represented by *g(x, y)*.

**Table 1:** Microarray data decision table

**Samples**	**Condition attributes (genes)**				**Decision attributes (classes)**
	**Gene 1**	**Gene 2**	**...**	**Gene *n***	**Class label**
1	*g*(1,1)	*g*(1,2)	...	*g*(1, *n*)	*Class (1)*

2	*g*(2,1)	*g*(2,2)	...	*g*(2, *n*)	*Class (2)*

...	...	...	...	...	...

*m*	*g*(*m*,1)	*g*(*m*,2)	...	*g*(*m*, *n*)	*Class (m)*

In rough sets, the *degree of dependency *of a set of attributes *Q *on another set of attributes *P *is denoted by *γ*_*P*_(*Q*) and is defined as



Where  represents the size of the union of the lower approximation of each equivalence class in *U*/*R*(*Q*) on *P *in *U*, and |*U*| represents the size of *U *(set of samples).

If *Q *is the decision attribute *D*, and *P *is a subset of condition attributes, then *γ*_*P*_(*D*) represents the *depended degree *of the condition attribute subset *P *by the decision attribute *D*; that is, to what degree *P *can discriminate the distinct classes of *D*. In this sense, *γ*_*P*_(*D*) reflects the classification power of the subset *P *of attributes. The greater is *γ*_*P*_(*D*), the stronger the classification ability *P *is inclined to possess. We chose the measure of the depended degree of condition attributes by class attributes as the basis for selecting informative genes.

In contrast to other correlation-based feature selection standards such as t-score, the *depended degree *can be calculated only when the attribute values are discrete. Thus, for the studied microarray datasets, the discretization of gene expression values is an essential step. Indeed, the discretization will bring about several advantages. First, some unimportant genes will be found immediately after the discretization. When the discretized expression values of a gene are identical among all of the samples, we view the gene as being insignificant because distinct classes cannot be separated according to the gene's expression values. Second, when gene expression values are reduced to discrete states, the rules formed by the genes can be described naturally via the discretized data.

However, for some datasets it is difficult to detect the discriminative features based on the depended degree because of its excessively rigid definition. In this case, we employed the *α *depended degree proposed in [17] as the basis for choosing genes. The *α depended degree *of an attribute subset *P *by the decision attribute *D *is defined as , where 0 ≤ *α *≤ 1,  and *pos(P, X, α) *= ∪{*Y *∈ *U/R*(*P*) | |*Y *∩ *X*|/|*Y*|≥ *α*} [17]. In fact, as indicated in [17], the depended degree is a specific case of the *α *depended degree when *α *= 1. In the case that the depended degree was largely ineffective as a basis on which to screen features, we employed the *α *(0.7 ≤ *α *< 1) depended degree.

Inducing decision rules that are hiding in decision tables is one of the key tasks of rough sets, which is also an essential procedure of our classifier construction. One decision rule in the form of "A ⇒ B" indicates that "if *A*, then *B*," where *A *is the description of condition attributes and *B *the description of decision attributes. The *confidence *of a decision rule *A *∧ *B *is defined as follows: , where support(*A*) denotes the proportion of the samples satisfying *A *and where support(*A *∧ *B*) denotes the proportion of the samples satisfying *A *and *B *simultaneously. The confidence of a decision rule indicates the reliability of the rule. If a decision rule had 100% confidence, we called it a *consistent decision rule*. It is evident that if *γ*_*P*_(*D*) equals 1, *P *⇒ *D *must be a consistent decision rule. In contrast, *γ*_*P*_(*D*, *α*) = 1 does not mean that *P *⇒ *D *must be a consistent decision rule.

To ensure the reliability of the classification rules, we chose only the genes or gene pairs with *γ*_*P*_(*D*) or *γ*_*P*_(*D*, *α*) equal to 1 when forming decision rules. Suppose *g *is one of the selected genes and *U *is the sample set. *U/R*(*g*) = {c_1_(*g*), c_2_(*g*), ..., c_n_(*g*)} represents the set of the equivalence class of samples induced by *R*(*g*). Two samples, s_1 _and s_2_, belong to the same equivalence class of *U/R*(*g*) if and only if they have the same value on *g*. In addition, we represented the set of the equivalence class of samples induced by *R*(*D*) as *U/R*(*D*) = {d_1_(*D*), d_2_(*D*), ..., d_m_(*D*)}, where *D *is the decision attribute. Likewise, two samples, s_1 _and s_2_, belong to the same equivalence class of *U/R*(*D*) if and only if they have the same value on *D*. For each c_i_(*g*) (i = 1, 2, ..., n), if there exists some d_j_(*D*) (j ∈ {1, 2, ..., m}), satisfying c_i_(*g*) ⊆ d_j_(*D*) in light of the depended degree or |c_i_(*g*) ∩ d_j_(*D*)|/|c_i_(*g*)|≥ *α *in light of the *α *depended degree, we then generated the following classification rule: *A*(c_i_(*g*)) ⇒ *B*(d_j_(*D*)), where *A*(c_i_(*g*)) is the formula describing the sample set c_i_(*g*) by the *g *value, and *B*(d_j_(*D*)) is the formula describing the sample set d_j_(*D*) by the class value. We used the same strategy to construct classification rules for gene pairs.

In the case of the depended degree, each employed classification rule was the consistent decision rule. However, in the case of the *α *depended degree, the classification rules may not have been consistent, yet the confidence of every classification rule must be no less than *α*, as proven in [17]. Hence, if we specified a large enough *α *threshold, the confidence of classification rules would have been sufficiently high.

### Datasets

#### Leukemia dataset 1 (ALL vs. AML)

The first dataset we analyzed was the well-known leukemia data studied by Golub et al. [2], which has been explored widely by many researchers. In this dataset, there are 72 observations, each of which is described by the gene expression levels of 7129 genes and a class attribute with two distinct labels: AML vs. ALL. The 72 observations are divided into a training set with 38 samples (27 ALL, 11 AML) and a test set with 34 samples (20 ALL, 14 AML).

#### Lung Cancer dataset

The Lung Cancer dataset is a classification of malignant pleural mesothelioma (MPM) vs. adenocarcinoma (ADCA) of the lung [15], and consists of 181 tissue samples (31 MPM, 150 ADCA). The training set contains 32 of the samples (16 MPM vs. 16 ADCA); the remaining 149 samples are used for testing. Each sample is described by 12,533 genes.

#### Prostate Cancer dataset

The Prostate Cancer dataset is concerned with prostate tumor vs. normal classification. The training set contains 52 prostate tumor samples and 50 non-tumor prostate samples [18]; the total number of genes is 12,600. Two classes are denoted as "Tumor" and "Normal." The test set samples were from a different experiment and have a nearly 10-fold difference in overall microarray intensity compared with the training data. We made use of the test set provided by Kent Ridge Bio-medical Data Set Repository, which includes 25 tumor and 9 normal samples.

#### Breast Cancer dataset

This dataset is concerned with the prediction of patient outcome for breast cancer [19]. The training set contains 78 patient samples, 34 of which are from patients who had developed distant metastases within 5 years ("relapse"); the remaining 44 samples are from patients who remained healthy from the disease for an interval of at least 5 years after initial diagnosis ("non-relapse"). There are 12 relapse and 7 non-relapse samples in the test set, and the number of genes is 24,481.

#### Leukemia dataset 2 (ALL vs. MLL vs. AML)

This dataset is about subtype prediction for leukemia [20]. The training set contains 57 samples (20 ALL, 17 MLL, and 20 AML), while the testing set contains 15 samples (4 ALL, 3 MLL, and 8 AML). The number of genes is 12,582.

The gene number, class, training sample number and test sample number contained in the five datasets are listed in Table 2.

**Table 2:** Summary of the five gene expression datasets

**Dataset**	**# Original genes**	**Class**	**# Training samples**	**# Test samples**
Leukemia 1	7129	ALL/AML	38 (27/11)	34 (20/14)

Lung Cancer	12533	MPM/ADCA	32 (16/16)	149 (15/134)

Prostate Cancer	12600	Tumor/Normal	102 (52/50)	34 (25/9)

Breast Cancer	24481	relapse/non-relapse	78 (34/44)	19 (12/7)

Leukemia 2	12582	ALL/MLL/AML	57 (20/17/20)	15 (4/3/8)

### Data preprocessing

#### Normalization of attributes value

Because the training set samples and the test set samples in the prostate cancer dataset are from two different experiments, and because discrepancies in microarray intensity exist between the two sets of samples, we normalized both the training set and the test set. Suppose that the original expression level of gene *y *in sample *x *is *g*(*x*, *y*). Then, the normalized value of *g*(*x*, *y*) is , where max g(•, *y*) and min g(•, *y*) represent the maximum and the minimum expression levels of gene *y *in all of the samples, respectively. After normalization, all of the expression levels of the genes lie within the interval [-1, 1]. As a result, we can apply the rules induced in the training set to the test set. Because the training set samples and the test set samples in the other datasets are from the same experiments, we chose not to normalize these data to avoid any loss of information.

#### Discretization of decision tables

Because rough sets is suitable for handling discrete attributes, we needed to first discretize the training set decision tables. We used the entropy-based discretization method, as first proposed by Fayyad et al. [21]. This algorithm recursively applies an entropy minimization heuristic to discretize the continuous-valued attributes. The stop of the recursive step for this algorithm depends on the minimum description length (MDL) principle. We implemented the discretization in the Weka package [22]. After the discretization, the majority of attributes contained at most two distinct values, while a small number of attributes contained three or four distinct values. We executed our learning algorithm in the discretized decision tables.

#### Feature selection, classifier construction, and validation

For the Leukemia 1 and Lung Cancer datasets, we conducted feature selection by the depended degree, while for the Prostate Cancer, Breast Cancer and Leukemia 2 datasets, we implemented feature selection by the *α *depended degree. For each dataset, we employed the LOOCV approach for the training set to identify high class-discrimination genes or gene pairs. That is, in the training set containing *n *samples, each sample is left out in turn, and the learning algorithm is trained on the remaining *n-1 *samples. Then, the training result is tested on the left-out sample. The final estimate is the average of *n *test results. We emphasize that only the single genes or gene pairs chosen by all of the leave-one-out training sets are used for LOOCV. In other words, when the depended degree standard is utilized, only those genes or gene pairs with a 100% depended degree in all leave-one-out training sets are selected; when the *α *depended degree standard is used, only the genes and gene pairs satisfying *γ*_*P*_(*D*, *α*) = 1 in all of the leave-one-out training sets are chosen. According to the results of LOOCV, we finally determined the informative genes or gene pairs. Applying the classification rules induced by the single genes or gene pairs in the entire training set to classify the independent test set, we further verified their classification performance.

## Results

### Classification results

#### Leukemia dataset 1

In this dataset, we first selected informative single genes. Among the 7129 genes, only gene #4847 had a 100% depended degree in all leave-one-out training sets. We denoted the expression level of gene x by g(x). The decision rules induced by gene #4847 in every leave-one-out training set are of the following form: if g(#4847) > t, then AML; if g(#4847) ≤ t, then ALL, where t is equal or close to 994. One can apply the decision rules to classify the left-out sample. The final LOOCV accuracy resulting from the gene was 97.4%, with 37 of the 38 samples classified correctly, wherein all of the 27 ALL samples were classified correctly, and one AML sample was misclassified. Subsequently, we examined the depended degree of the gene in the whole training set of 38 samples. As expected, the gene had a 100% depended degree in the training set. The two consistent decision rules generated by this gene were as follows: if g(#4847) > 994, then AML; if g(#4847) ≤ 994, then ALL. One can use the above rules to classify the independent test set with 91.2% classification accuracy. Among the 34 samples, 31 were classified correctly and 3 were classified incorrectly: 2 ALL samples were misclassified into AML, and 1 ALL sample was misclassified into AML.

Next, we searched for informative gene pairs. Because there are 7129 genes, the combination number would be huge if all were taken into account. Therefore, for each leave-one-out training set, only the genes with more than 18/37 depended degree were considered in forming gene pairs (excluding the aforementioned gene #4847). As a result, 350 gene pairs were found to possess a 100% depended degree in all leave-one-out training sets. Every gene pair was capable of inducing four consistent decision rules, which were used for classification. We set the threshold of LOOCV accuracy such that at least 35 of the 38 samples were classified correctly. Accordingly, 347 gene pairs satisfied the condition. Likewise, using the decision rules induced by the gene pairs in the whole training set to classify the test set, we detected 13 gene pairs with no less than 32 test samples classified correctly (at most, 2 errors). Table 3 lists data for these 13 pairs of genes. In this table, the classification results regarding LOOCV and the test set are shown in terms of both the number of correctly classified samples and accuracy. The results with respect to every class are presented in parentheses, and the optimal results are formatted in boldface.

**Table 3:** Thirteen gene pairs with high classification accuracy in the Leukemia dataset 1

**1st - 2nd Probe ID**	**Classification results in LOOCV**		**Classification results in the test set**	
	
	**# Correctly classified samples**	**Accuracy (%)**	**# Correctly classified samples**	**Accuracy (%)**
U46499_at - M92287_at	35 (26/9)	92.11 (96.30/81.82)	33 (20/13)	97.06 (100/92.86)

U46499_at - M12959_s_at	36 (27/9)	94.74 (100/81.82)	**34 **(20/14)	**100 **(100/100)

U46499_at - D63880_at	36 (27/9)	94.74 (100/81.82)	33 (20/13)	97.06 (100/92.86)

U46499_at - S50223_at	**37 **(27/10)	**97.37 **(100/90.91)	33 (19/14)	97.06 (95/100)

U46499_at - Z15115_at	35 (26/9)	92.11(96.30/81.82)	33 (20/13)	97.06 (100/92.86)

L09209_s_at - M92287_at	**37 **(27/10)	**97.37 **(100/90.91)	33 (20/13)	97.06 (100/92.86)

L09209_s_at - S50223_at	**37 **(27/10)	**97.37 **(100/90.91)	33 (19/14)	97.06 (95/100)

X61587_at - M92287_at	36 (26/10)	94.74 (96.30/90.91)	33 (20/13)	97.06 (100/92.86)

X61587_at - M12959_s_at	**37 **(27/10)	**97.37 **(100/90.91)	33 (19/14)	97.06 (95/100)

L09209_s_at - D63880_at	**37 **(27/10)	**97.37 **(100/90.91)	32 (19/13)	94.12 (95/92.86)

U05259_rna1_at - M92287_at	36 (26/10)	94.74 (96.30/90.91)	32 (20/12)	94.12 (100/100)

L09209_s_at - X59417_at	**37 **(27/10)	**97.37 **(100/90.91)	32 (19/13)	94.12 (95/92.86)

L09209_s_at - Z15115_at	**37 **(27/10)	**97.37 **(100/90.91)	32 (19/13)	94.12 (95/92.86)

Among the 13 gene pairs, the combination #3252-#6167 possessed 100% classification accuracy on the test set. The decision rules produced by the gene pair were as follows:

• if g(#3252) ≤ 156.5 and g(#6167) > 820.5, then ALL;

• if g(#3252) ≤ 156.5 and g(#6167) ≤ 820.5, then ALL;

• if g(#3252) > 156.5 and g(#6167) > 820.5, then ALL;

• if g(#3252) > 156.5 and g(#6167) ≤ 820.5, then AML.

The above rules were then simplified into three equivalent rules:

• if g(#3252) ≤ 156.5, then ALL;

• if g(#6167) > 820.5, then ALL;

• if g(#3252) > 156.5 and g(#6167) ≤ 820.5, then AML.

These three rules are fairly simple and easily understood. Using these rules, we classified the test set without any errors. The rules derived from the other 12 gene pairs are provided in the Additional file 1, and we also provide information on the top 87 genes in the training set with depended degrees of no less than 0.5 in the Additional file 2.

#### Lung Cancer dataset

This dataset contained 16 genes with a 100% depended degree in all of the 32 leave-one-out training sets. The LOOCV accuracy of the 16 genes was between 93.75% and 100%. Namely, the number of correctly classified samples ranged from 30 to 32. In the training set, each of the 16 genes had a 100% depended degree. These observations indicate that each single gene among the 16 genes was likely to have high class-discriminative power in the training set. Using the rules generated by these single genes, we examined the test set. As expected, these genes showed high classification performance, with classification accuracy ranging from 79% to 97%. The classification results are presented in Table 4, which shows that some of the genes in the Lung Cancer dataset, such as gene 37716_at, have impressive classification performance. The rules induced by gene 37716_at were the following: if g(37716_at) > 197.75, then mesothelioma; if g(37716_at) ≤ 197.75, then ADCA. Using these two rules, we could classify the test set with 97% accuracy. The rules produced by the 16 genes are provided in the Additional file 3. From these rules, we suspected that 2047_s_at, 2266_s_at, 32046_at, 33245_at, 41286_at, 41402_at, 575_s_at, and 988_at have higher expression levels in ADCA, while the others have higher expression levels in mesothelioma.

**Table 4:** Sixteen genes with high classification accuracy in the Lung Cancer dataset

**Probe ID**	**Classification results in LOOCV**		**Classification results in the test set**	
	
	**# Correctly classified samples**	**Accuracy(%)**	**# Correctly classified samples**	**Accuracy(%)**
2047_s_at	30 (15/15)	93.75 (93.75/93.75)	122 (11/111)	81.88 (73.33/82.84)

266_s_at	**32 **(16/16)	**100 **(100/100)	129 (13/116)	86.58 (86.67/86.57)

32046_at	30 (15/15)	93.75 (93.75/93.75)	133 (12/121)	89.26 (80/90.30)

32551_at	31 (15/16)	96.88 (93.75/100)	134 (14/120)	89.93 (93.33/89.55)

33245_at	30 (15/15)	93.75 (93.75/93.75)	137 (14/123)	91.95 (93.33/91.79)

33833_at	**32 **(16/16)	**100 **(100/100)	139 (13/126)	93.29 (86.67/94.03)

35330_at	31 (15/16)	96.88 (93.75/100)	118 (14/104)	79.19 (93.33/77.61)

36533_at	30 (15/15)	93.75 (93.75/93.75)	141 (13/128)	94.64 (86.67/95.52)

37205_at	30 (15/15)	93.75 (93.75/93.75)	135 (12/123)	90.60 (80/91.79)

37716_at	30 (15/15)	93.75 (93.75/93.75)	**145 **(11/134)	**97.32 **(73.33/100)

39795_at	31 (16/15)	96.88 (100/93.75)	135 (14/121)	90.60 (93.33/90.30)

40936_at	31 (15/16)	96.88 (93.75/100)	140 (12/128)	93.96 (80/95.52)

41286_at	30 (15/15)	93.75 (93.75/93.75)	121 (13/108)	81.21 (86.67/80.60)

41402_at	31 (16/15)	96.88 (100/93.75)	123 (13/110)	82.55 (86.67/82.09)

575_s_at	**32 **(16/16)	**100 **(100/100)	141 (14/127)	94.64 (93.33/94.78)

988_at	30 (15/15)	93.75 (93.75/93.75)	132 (13/119)	88.59 (86.67/88.81)

If more than one gene is considered when developing rules, higher classification accuracy should be achieved. Therefore, we carried out further classification tests using gene pairs. As before, we tried to find the gene pairs with high LOOCV accuracy. To avoid combination explosion, to constitute gene pairs we only selected genes with more than 12/31 and less than 100% depended degree in all 32 leave-one-out training sets. Furthermore, to avoid intricate classification rules produced by gene pairs, we excluded genes with more than two distinct discretized values. Accordingly, we found 82 gene pairs with a 100% depended degree in all 32 leave-one-out training sets. Among them, 25 pairs possessed 100% LOOCV accuracy. These pairs also had comparatively strong classification power in the test set. Their classification accuracy was between 71.14% and 96.64%; 21 pairs showed accuracy exceeding 80%, and nine pairs had accuracy exceeding 90%. Data for these 25 gene pairs are listed in Table 5. The classification rules induced by these pairs are presented in the Additional file 3.

**Table 5:** Twenty-five gene pairs with 100% LOOCV accuracy in the Lung Cancer dataset

**1st - 2nd Probe ID**	**Classification results in the test set**	
	
	**# Correctly classified samples**	**Accuracy (%)**
33754_at - 36562_at	**144 **(13/131)	**96.64 **(86.67/97.76)

33754_at - 40496_at	143 (11/132)	95.97 (73.33/98.51)

34105_f_at - 40496_at	141(9/132)	94.64 (60/98.51)

34105_f_at - 36562_at	140 (10/130)	93.96 (66.67/97.01)

37004_at - 40496_at	140 (11/129)	93.96 (73.33/96.27)

36562_at - 37004_at	139 (13/126)	93.29 (86.67/94.03)

38827_at - 40445_at	138 (15/123)	92.62 (100/91.79)

1882_g_at - 36562_at	136 (11/125)	91.28 (73.33/93.28)

1882_g_at - 40496_at	136 (10/126)	91.28 (66.67/94.03)

33907_at - 36562_at	134 (10/124)	89.93 (66.67/92.54)

36562_at - 40496_at	134 (9/125)	89.93 (60/93.28)

1882_g_at - 33907_at	133 (11/122)	89.26 (73.33/91.04)

1882_g_at - 37004_at	132 (13/119)	88.59 (86.67/88.81)

35947_at - 36269_at	132 (12/120)	88.59 (80/89.55)

33907_at - 34105_f_at	131(9/122)	87.92 (60/91.04)

36269_at - 40445_at	131(14/117)	87.92 (93.33/87.31)

35947_at - 40445_at	130 (14/116)	87.25 (93.33/86.57)

38074_at - 38827_at	129 (14/115)	86.58 (93.33/85.82)

33907_at - 40496_at	127(8/119)	85.23 (53.33/88.81)

36269_at - 38074_at	125 (13/112)	83.89 (86.67/83.58)

38074_at - 40445_at	122 (13/109)	81.88 (86.67/81.34)

1117_at - 38827_at	116 (15/101)	77.85 (100/75.37)

1117_at - 36269_at	113 (13/100)	75.84 (86.67/74.63)

1117_at - 35947_at	109 (12/97)	73.15 (80/72.39)

1117_at - 38074_at	106 (14/92)	71.14 (93.33/68.66)

To observe the relationship between the depended degrees of single genes and the classification accuracy of gene pairs, we carried out another experiment. In the discretized training set, we first excluded the genes with depended degrees 0 and 100%, as well as the genes with above two distinct values. As a result, there were 1428 genes left for pair combination. We set the threshold number of correctly classified samples as 148; that is, we searched for the gene pairs by which the test set are classified with at most one error. In addition, we set another threshold *k*, and required that the sizes of the positive regions caused by the selected genes must exceed *k*, with *k *varying from 13 to 0. When *k *equals 13, 61 genes are selected, and 743 pair combinations have 100% depended degree. Using the rules derived from each of the 743 gene pairs to classify the test set, we detected 4 combinations with 148 samples classified correctly. When *k *was 12, 11, and 10, only the same four combinations were found. When *k *decreased to 9 and 8, five and seven combinations were found, respectively. At lower values, no more combinations were found to classify 148 samples or more correctly, even when *k *was reduced to 0, and the selected gene number is 1428 accompanied by 33,390 combinations with a 100% depended degree. The results indicate that combinations between genes with higher depended degrees are more likely to produce accurate classification.

To explore whether the combinations between the genes with 100% depended degrees and other genes with lower depended degrees would yield more gene pairs having no less than 148 samples classified correctly, we added the 16 genes with a 100% depended degree to the 1428 genes and repeated the above experiment. Surprisingly, the results were exactly the same as those of the first experiment; i.e., no new gene pair was found. This finding indicates that to obtain perfect classification performance by combined genes, although the class-discrimination ability of individual genes is important, the mutual information complement between individual genes might also be crucial. Additional details regarding this experiment are provided in Table S1 of the Additional file 4.

Table S2 of the Additional file 4 shows the most seven pair combinations found in the experiment. Each of the seven gene pairs generates four rules, which can be simplified into three equivalent rules. The rules can be used to correctly classify 148 of 149 samples in the test set, with only one error (one mesothelioma was misclassified as ADCA). The detailed rules formed by the seven pairs of genes are presented in the Additional file 3.

#### Prostate Cancer dataset

Because of differences in microarray intensity between the training set and the test set, we first normalized the attribute values for both sets. Every attribute value was normalized to a number between -1 and 1. In this dataset, if the depended degree standard is employed for gene selection, it is somewhat difficult to find authentically discriminative genes, as no gene has a 100% depended degree, and the highest depended degree in the training set is 36%. Therefore, we utilized the *α *depended degree as the criterion for gene selection. For *α *≥ 0.9, no common gene was detected among all of the 102 leave-one-out training sets; when *α *= 0.85, gene #10493 was found; when *α *= 0.80, nine genes were found. Of these nine genes, we excluded gene #5261 with three distinct values, and calculated the LOOCV accuracy of the other eight genes. Relatively high LOOCV outcomes were obtained. Applying the decision rules induced by each of the eight genes in the training set, we classified the test set and achieved satisfactory classification results (see Table 6). The classification rules generated by the eight genes are presented in the Additional file 5.

**Table 6:** Eight genes with high classification accuracy in the Prostate Cancer dataset

**Probe ID**	**Classification results in LOOCV**		**Classification results in the test set**		***α***
		
	**# Correctly classified samples**	**Accuracy (%)**	**# Correctly classified samples**	**Accuracy (%)**	
32598_at	**92 **(50/42)	**90.20 **(96.15/84.00)	23 (17/6)	67.65 (68.00/66.67)	0.85

36491_at	84 (41/43)	82.35 (78.85/86.00)	30 (23/7)	88.24 (92.00/77.78)	0.80

40856_at	85 (46/39)	83.33 (88.46/78.00)	23 (15/8)	67.65 (60.00/88.89)	0.80

32243_g_at	84 (41/43)	82.35 (78.85/86.00)	**31 **(22/9)	**91.18 **(88.00/100)	0.80

36601_at	85 (46/39)	83.33 (88.46/78.00)	17 (8/9)	50.00 (32.00/100)	0.80

38044_at	81 (41/40)	79.41 (78.85/80.00)	29 (21/8)	85.29 (84.00/88.89)	0.80

41288_at	88 (41/47)	86.27 (78.85/94.00)	**31 **(22/9)	**91.18 **(88.00/100)	0.80

1767_s_at	83 (40/43)	81.37 (76.92/86.00)	24 (22/2)	70.59 (88.00/22.22)	0.80

As for gene pairs, when *α *= 0.75 and the threshold of the positive region sizes caused by single genes was 13, 16 gene pairs were shared by all 102 of the leave-one-out training sets. The LOOCV accuracy of the 16 gene pairs was between 81% and 86%, yet there were three pairs of genes with relatively good classification performance in the test set (Table 7). The classification rules generated by the three pairs are presented in the Additional file 5.

**Table 7:** Three gene pairs with good classification accuracy in the Prostate Cancer dataset

**1st - 2nd Probe ID**	**Classification results in LOOCV**		**Classification results in the test set**		***α***
		
	**# Correctly classified samples**	**Accuracy (%)**	**# Correctly classified samples**	**Accuracy (%)**	
35178_at - 35277_at	83 (33/50)	81.37 (63.46/100)	26 (20/6)	76.47 (80.00/66.67)	0.75

35178_at - 38087_s_at	83 (33/50)	81.37 (63.46/100)	**27 **(21/6)	**79.41 **(84.00/66.67)	0.75

39331_at - 33121_g_at	**86 **(38/48)	**84.31 **(73.08/96.00)	**27 **(18/9)	**79.41 **(72.00/100)	0.75

We also analyzed the training set based on the depended degree. We ranked all of the genes in the discretized training set by their depended degrees. The top two genes, 37639_at and 41755_at, had the highest depended degree of 36%. When we examined the rules formed by gene 37639_at, we found the following: if g(37639_at) > -0.491443, then Tumor (100% confidence); if g(37639_at) ≤ -0.694377, then Normal (95% confidence). Both rules were highly reliable. Using the two rules, we correctly classified 33 of the 34 test samples. This result indicates that gene 37639_at possessed high class-discrimination power. The rules arising from this gene indicate that it is relatively highly expressed in tumor samples. Gene 41755_at produced the following two rules: if g(41755_at) > 0.261438, then Tumor (100% confidence); if g(41755_at) ≤ -0.477124, then Normal (100% confidence). Using these two rules, 14 of the 34 test samples were classified correctly, whereas all 9 samples labeled "Normal" were classified correctly. The rules implied that gene 41755_at is expressed at a low level in normal samples. Apart from 37639_at and 41755_at, gene 38087_s_at produced the following rule: if g(38087_s_at) > -0.281725, then Normal (100% confidence). We correctly classified six of nine normal samples using the rule, indicating that this gene is comparatively highly expressed in normal samples. Information on the top 20 genes ranked based on depended degree is provided in the Additional file 6.

#### Breast Cancer dataset

In the dataset, when *α *≥ 0.8, no shared gene was detected in all of the 78 leave-one-out training sets; when *α *= 0.75, four genes were found; when *α *= 0.70, 46 genes were found. Most of these 46 genes had LOOCV accuracy ranging from 70% to 80%, while a few had LOOCV accuracy slightly less than 70%. Using each of the 46 genes to classify the test set, we found eight genes by which no less than 13 of the 19 test samples were classified correctly. Information on the eight genes is listed in Table 8. The classification rules generated by each of the eight genes are available in the Additional file 7. In the dataset, we did not find any gene pairs with satisfactory classification performance. The best classification accuracy obtained by gene pairs was 12 test samples classified correctly; accuracy was 63.16%.

**Table 8:** Eight genes with high classification accuracy in the Breast Cancer dataset

**GenBank accession number**	**Classification results in LOOCV**		**Classification results in the test set**		***α***
		
	**# Correctly classified samples**	**Accuracy (%)**	**# Correctly classified samples**	**Accuracy (%)**	
NM_012261	57 (21/36)	73.08 (61.76/81.82)	**16 **(10/6)	**84.21 **(83.33/85.71)	0.70

AW237580	**58 **(18/40)	**74.36 **(52.94/90.91)	13 (8/5)	68.42 (66.67/71.43)	0.70

U45975	**58 **(22/36)	**74.36 **(64.71/81.82)	13 (9/4)	68.42 (75.00/57.14)	0.70

AI742029	55 (17/38)	70.51 (50.00/86.36)	13 (11/2)	68.42 (91.67/28.57)	0.70

NM_001689	57 (22/35)	73.08 (64.71/79.55)	15 (9/6)	78.95 (75.00/85.71)	0.70

TSPYL5	**58 **(24/34)	**74.36 **(70.59/77.27)	**16 **(10/6)	**84.21 **(83.33/85.71)	0.70

NM_000271	57 (20/37)	73.08 (58.82/84.09)	13 (9/4)	68.42 (75.00/57.14)	0.70

AL049689	55 (22/33)	70.51 (64.71/75.00)	13 (10/3)	68.42 (83.33/42.86)	0.70

#### Leukemia dataset 2

This dataset contains three classes, being a multi-class classification problem. When *α *≥ 0.95, no shared gene was detected in the 57 leave-one-out training sets; when *α *= 0.9 and 0.85, a single gene was found; when *α *= 0.80, five genes were found; when *α *= 0.75, eight genes were found; when *α *= 0.70, 21 genes were identified. Almost every one of these 21 genes had a high LOOCV accuracy and good classification performance in the test set. Their classification information is listed in Table 9. Gene 36239_at had the best LOOCV accuracy and classification accuracy in the test set. The classification rules induced by this gene were as follows: if g(36239_at) > 1796.5, then ALL; if g(36239_at) > 214 and g(36239_at) ≤ 1796.5, then MLL; if g(36239_at) ≤ 214, then AML; with 95.24%, 93.33%, and 90.48% confidence, respectively. Using these three rules, we correctly classified 14 of the 15 test samples; accuracy reached 93.33%. The other genes produced similar classification rules. The classification rules generated by every gene can be found in the Additional file 8. We did not examine gene pairs for the classification, as the rules induced by gene pairs tended to be complex.

**Table 9:** Twenty-one genes with high classification accuracy in the Leukemia dataset 2

**Probe ID**	**Classification results in LOOCV**		**Classification results in the test set**		***α***
	**# Correctly classified samples**	**Accuracy (%)**	**# Correctly classified samples**	**Accuracy (%)**	

36239_at	**51 **(20/12/19)	**89.47 **(100/70.59/95)	**14 **(4/2/8)	**93.33 **(100/66.67/100)	0.90

39318_at	47 (17/11/19)	82.46 (85/64.71/95)	13 (2/3/8)	86.67 (50/100/100)	0.80

40191_s_at	48 (17/13/18)	84.21 (85/76.47/90)	12 (2/2/8)	80 (50/66.67/100)	0.80

840_at	47 (19/10/18)	82.46 (95/58.82/90)	11 (3/1/7)	73.33 (75/33.33/87.50)	0.80

266_s_at	46 (19/11/16)	80.70 (95/64.71/80)	13 (4/1/8)	86.67 (100/33.33/100)	0.80

37933_at	45 (20/7/18)	78.95 (100/41.18/90)	8 (2/0/6)	53.33 (50/0/75)	0.75

38989_at	43 (19/6/18)	75.44 (95/35.29/90)	12 (3/1/8)	80 (75/33.33/100)	0.75

33833_at	44 (16/10/18)	77.19 (80/58.82/90)	10 (2/0/8)	66.67 (50/0/100)	0.75

32874_at	43 (14/11/18)	75.44 (70/64.71/90)	10 (2/1/7)	66.67 (50/33.33/87.5)	0.7

37487_at	41 (14/7/20)	71.93 (70/41.18/100)	11 (3/0/8)	73.33 (75/0/100)	0.7

31886_at	42 (16/8/18)	73.68 (80/47.06/90)	13 (3/2/8)	86.67 (75/66.67/100)	0.7

35164_at	48 (19/15/14)	84.21 (95/88.24/70)	13 (4/2/7)	86.67 (100/66.67/87.5)	0.7

36905_at	46 (14/12/20)	80.70 (70/70.59/100)	9 (0/1/8)	60 (0/33.33/100)	0.7

37539_at	50 (16/16/18)	87.72 (80/94.12/90)	10 (3/3/4)	66.67 (75/100/50)	0.7

37910_at	45 (18/9/18)	78.95 (90/52.94/90)	9 (1/1/7)	60 (25/33.33/87.5)	0.7

32847_at	44 (18/12/14)	77.19 (90/70.59/70)	11 (4/2/5)	73.33 (100/66.67/62.5)	0.7

35260_at	42 (20/8/14)	73.68 (100/47.06/70)	9 (2/1/6)	60 (50/33.33/75)	0.7

41790_at	47 (19/11/17)	82.46 (95/64.71/85)	13 (3/2/8)	86.67 (75/66.67/100)	0.7

32579_at	48 (15/13/20)	84.21 (75/76.47/100)	11 (2/1/8)	73.33 (50/33.33/100)	0.7

1373_at	47 (16/12/19)	82.46 (80/70.59/95)	10 (1/1/8)	66.67 (25/33.33/100)	0.7

1325_at	47 (19/14/14)	82.46 (95/82.35/70)	10 (3/3/4)	66.67 (75/100/50)	0.7

### Comparison and analysis of results

#### Leukemia dataset 1

Other researchers have explored the problem concerned with the classification of the dataset using rule-based machine-learning methods. In [7], the authors proposed first using feature ranking (t-test) and then rough sets attribute reduction for gene selection. They ultimately identified one gene, which classified 31 samples correctly in the test set. This gene was the gene identified in the present study: gene #4847. However, our method identified not only this gene, but also other informative genes, including one gene pair with 100% classification accuracy. In [8], the authors also used rough sets for gene selection. They chose genes with maximum relevance with respect to the class variable and the maximum positive interaction between different genes. We also selected genes with maximum relevance with respect to the class variable (i.e., the depended degree of a single gene), while we chose gene pairs with maximum relevance with respect to the class variable rather than maximum positive interaction between the genes, since the maximum positive interaction between two genes may counteract the depended degree of a single gene. Because this previous study assessed classification performance using LOOCV on a total of 72 samples instead of separating them into training and test sets, it is impractical to compare their results with those of the present study. Likewise, in [9] the authors took into account all attributes depending upon the degree of dependency. They selected the top *λ *attributes (*λ *= 2, 4, 6, 8, 10, 12, 14, 15) by the degree of dependency, and found all possible combinations of these *λ *attributes as a subset. The authors calculated the depended degrees of every subset and chose those with 100% depended degrees. Finally, they evaluated the classification performance of the selected subsets using *k*-NNs. In essence, their method was to find the reducts with limited sizes and to use them for classification. As we mentioned above, finding all of the reducts is computationally intensive, even for a small attribute number. Moreover, one reduct does not indicate high classification performance. Another difference between our method and that of [9] is that our classifier is based on rules, whereas theirs is not. Although they gain a classification score of 97% with gene subsets of size two, they did not find any gene pair with a classification score of 100%, and they did not identify any important genes. In [10], a method of combining rough sets with GAs was proposed to classify microarray gene expression patterns. A correct classification of 90.3% was obtained with a nine-gene classifier in the dataset.

In [6], the authors used the EPs approach to mark one important gene, Zyxin, which is our gene #4847. Using the two rules induced by the gene, the authors accurately classified 31 samples, the same result as ours. However, they did not identify any gene pair with higher classification performance, as we did. In [11], the authors used decision trees (Single C4.5, Bagging C4.5, AdaBoost C4.5) to perform classification tasks on seven publicly available cancerous microarray datasets, including the ALL-AML leukemia data. They first employed Fayyad and Irani's [21] discretization method to filter out noise. The remaining 1038 genes were used in the actual learning process. Their highest accuracy was 91.2% (31 samples classified correctly). Since the authors did not report the size of the pruned decision trees, we have no knowledge of how many genes they used to reach the highest accuracy. In [13], 91.2% classification accuracy was achieved by using the rule classifiers containing gene subsets with sizes ranging from 10 to 40. In [14], the authors utilized a single pair of genes to correctly classify 31 test set samples.

Besides, a number of different non-rule-based methods have been proposed for gene selection and cancer classification in the dataset. Golub et al. [2] were the first to classify ALL-AML by gene expression data. The authors constructed the predictor using 50 informative genes, trained by weighted voting on the training set. The prediction rates included 36 samples classified correctly, with two samples labeled "uncertain" in LOOCV, as well as 29 of the 34 samples in the test set classified correctly, with no predictions made for the remaining five samples. In [23], the authors applied probabilistic neural networks (PNNs) to the class prediction of ALL-AML, and achieved 100% prediction accuracy in the test set using the 50-gene predictors derived from cross-validation tests of the training set by means of the signal-to-noise statistic feature selection method. In [24], the authors used a correlation-based feature (CBF) selector in conjunction with machine-learning algorithms such as decision trees (JP48), NB, and SVMs to analyze cancer microarray data. They reported one noteworthy gene, Zyxin, which classified 31 samples correctly. In [25], the authors proposed a maximal margin linear programming (MAMA) method for the classification of tumor samples based on microarray data. This procedure detected groups of genes and constructed models that strongly correlated with particular tumor types. They achieved 100% prediction accuracy on the test set using gene subsets ranging in size from 132 to 549. In [26], the authors proposed dimension reduction using partial least squares (PLS) and classification using logistic discrimination (LD) and quadratic discriminant analysis (QDA). By using gene subsets with sizes between 50 and 1500, the authors obtained correct classification of the test samples ranging from 28 to 33. In [27], the authors used SVMs trained and gene subsets selected in the training set to classify samples in the test set, resultng in the correct classification of between 30 and 32 of the 34 samples. Other SVM-based methods report zero test error with gene subsets ranging in size from 8 to 30 [28-30].

Table 10 compares our methods with those employed in previous studies. The table reveals that our classification results are superior to almost all of those obtained in previous studies.

**Table 10:** Comparison of best classification accuracy for the Leukemia dataset 1

**Methods (feature selection + classification)**^a^	**#Selected genes**	**#Correctly classified samples (accuracy)**	**Rule-based classifier**
depended degree + decision rules [this work]	1	31 (91.18%)	yes
		
	2	34 (100%)	

t-test, attribute reduction + decision rules [7]	1	31 (91.18%)	yes

attribute reduction + *k*-NNs [9]	2	33 (97.06%)	no

rough sets, GAs + *k*-NNs [10]	9	31 (91.18%)	no

EPs [6]	1	31 (91.18%)	yes

discretization + decision trees [11]^b^	unknown^c^	31 (91.18%)	yes

CBF + decision trees [24]	1	31 (91.18%)	yes

TSP [14]	2	31 (91.18%)	yes

RCBT [13]	10-40	31 (91.18%)	yes

neighborhood analysis + weighted voting [2]	50	29 (85.29%)	no

signal to noise ratios + PNNs [23]	50	34 (100%)	no

MAMA [25]	132-549	34 (100%)	no

PLS + LD or QDA [26]	50-1500	28-33 (82.4%-97%)	no

prediction strength + SVMs [27]	25-1000	30-32 (88.2%-94.1%)	no

SVMs [28-30]	8-30	34 (100%)	no

^a^The text before "+" states the feature selection method, while that after it states the classification method. The absence of "+" means that the same method was used for both feature selection and classification.

^b^The decision trees are also involved in feature selection.

^c^"unknown" means that no related data are provided in the article.

These explanations apply to the other tables.

In this dataset, we identified 11 genes that show good classification performance alone or in combination with another gene. These genes are Zyxin, MGST1, TCRA, APLP2, CCND3, HKR-T1, KIAA0159, TOP2B, MB-1, ARHG, and IOTA. Among these, Zyxin, CCND3, HKR-T1, TOP2B, MB-1, and IOTA also belong to the list of the 50 informative genes identified by Golub et al. [2]; Zyxin is highly expressed in AML, and the rest are highly expressed in ALL. Our rules relevant to these genes revealed that Zyxin, MGST1, APLP2, and ARHG are upregulated in AML, while TCRA, CCND3, HKR-T1, KIAA0159, TOP2B, MB-1, and IOTA are upregulated in ALL. These results demonstrate that our rules are reasonable.

Our method identified an outstanding gene, Zyxin, by which we classified the test set with 91.2% accuracy. The gene is also referred to by other researchers [2,6,7,23,24,26,27,31-36]. Our results and those of other related studies suggest that the expression level of Zyxin plays an important role in distinguishing ALL from AML. Zyxin is a focal-adhesion-associated phosphoprotein with one domain involved in the control of actin assembly and three protein-protein adapter domains implicated in the regulation of cell growth and differentiation. Zyxin may function as a messenger in the signal transduction pathway that mediates adhesion-stimulated changes in gene expression. As noted in [36], cell spreading, proliferation, and survival are modulated by focal adhesions linking extracellular matrix proteins, integrins, and the cytoskeleton. By supporting the involvement of the microfilament network in tumor cell behavior, several actin-binding proteins, including Zyxin, a potential regulator of actin polymerization, may play a role in oncogenesis. The gene encoding Zyxin maps at 7q32, a chromosomal region affected in a variety of human cancers. 7q monosomy or partial deletion of this chromosome arm is frequently found in myelodysplastic syndrome, acute myeloid, juvenile myelomonocytic, and acute lymphocytic leukemias, as well as in breast carcinoma [37,38]. Valdes et al. revealed that the actin cytoskeleton-associated protein Zyxin acts as a tumor suppressor in Ewing tumor cells [32]. Yagi et al. also identified Zyxin as one of 35 genes associated with pediatric AML prognosis [31]. Taken together, these lines of evidence suggest that Zyxin plays an important role in leukemia pathogenesis.

The aforementioned gene pair, MGST1 vs. TCRA, is capable of classifying the test set with zero error. Their biological meanings are noteworthy. MGST1 is also one of the three core genes screened by Banerjee et al. [10]. In [24], the gene lies in the first 10 genes selected by the methods of *χ*^2^, InfoGain, ReliefF, and symmetrical uncertainty. In [23], MGST1 belonged to the set of top 50 genes selected by signal-to-noise metric (10-fold cross-validation tests). In our 13 gene pairs with the highest classification performance, MGST1 occurred five times. These facts demonstrate that MGST1 is significant in the classification of ALL-AML. Although it has not been identified by other algorithms, the gene TCRA is clearly important in the pathogenesis of leukemia [39-41].

APLP2 was one of the first 10 genes selected by Wang et al. [24], and was identified by Huang et al. [23]. It was also identified by Yagi et al. [31] as one of 35 genes associated with pediatric AML prognosis. CCND3 is also listed as one of the 50 genes selected by Huang et al. [23]. KIAA0159 is an essential component of the human condensin complex required for mitotic chromosome condensation. In a brief examination of related literature, we found that the gene has not been identified by other algorithms. However, past studies have indicated that nonrandom chromosomal translocations are characteristic of most human hematopoietic malignancies [42]. Because KIAA0159 is correlated with the structural maintenance of chromosomes, it may be associated with the pathogenesis of leukemia. TOP2B encodes the protein that is the principal target of the antileukemic drug etoposide [2,43,44]. MB-1 encodes the Ig-alpha protein of the B-cell antigen component. Its dysregulation has been reported to be closely linked to leukemia and lymphoma [45-48]. ARHG is a member of the RAS superfamily of genes, which encode GTP-binding proteins that act in the pathway of signal transduction and play a key role in the regulation of cellular functions [49].

In general, the genes identified in the present study are all directly or indirectly relevant to hematopoietic or cancerous pathogenesis. Therefore, they are likely to play key roles in the pathogenesis of ALL or AML. It is possible that they have high performance in distinguishing ALL from AML.

#### Lung Cancer dataset

In [9], the authors used rough sets to handle the same dataset as that considered in the present study. Their best result was 98% classification accuracy with genes of size two. As they employed a non-rule-based classifier, *k*-NN, no rule was given to explain the result. In [50], in terms of classification performance, the authors compared prediction by collective likelihoods (PCLs), based on the concept of EPs, with other classification algorithms, including decision trees, SVMs, and *k-*NNs. Regarding the Lung Cancer dataset, they obtained classification results containing between 1 and 27 errors. The classification accuracy of our method is higher than that of other rule-based classification algorithms, including PCLs and the decision trees mentioned in [50]. The highest classification accuracies on the dataset, using the three different decision trees reported in [11], were about 93%. In [13], the best result was 98% classification accuracy. In the initial research article on the dataset [15], the authors reported 99% classification accuracy using six genes. Table 11 compares our results with those of other studies, revealing that our outcomes matched or outperformed those obtained using other methods.

**Table 11:** Comparison of best classification accuracy for the Lung Cancer dataset

**Methods (feature selection + classification)**	**#Selected genes**	**#Correctly classified samples (accuracy)**	**Rule-based classifier**
depended degree + decision rules [this work]	1	145 (97.34%)	yes
		
	2	144 (96.64%)	

attribute reduction + *k*-NNs [9]	2	146 (97.99%)	no

PCLs [50]	unknown	146 (97.99%)	yes

C4.5 [50]	1	122 (81.88%)	yes

Bagging [50]	unknown	131 (87.92%)	yes

Boosting [50]	unknown	122 (81.88%)	yes

SVMs [50]	unknown	148 (99.33%)	no

*k*-NNs [50]	unknown	148 (99.33%)	no

discretization + decision trees [11]	unknown	139 (93.29%)	yes

RCBT [13]	10-40	146 (97.99%)	yes

gene expression ratios [15]	6	148 (99.33%)	no

We now explain in more detail the results presented in [15]. The article proposed to use the expression levels of a small number of genes for the diagnosis of MPM and lung cancer. The authors screened out eight genes with marked differences in average expression levels between the tumor types in the training set. They then calculated 15 expression ratios for each sample by dividing the expression value of each of the five genes expressed at relatively higher levels in MPM by the expression value of each of the three genes expressed at relatively higher levels in ADCA. Next, they employed these ratios for the test set. Samples with ratio values > 1 were classified as MPM, and those with ratio values < 1 were classified as ADCA. They achieved classification accuracies ranging from 91% to 98%. In essence, they also utilized gene pairs for classification. Yet, when following the same protocol for training and testing, our results are superior to theirs, in that they used three ratios (i.e., six genes) to reach 148 of 149 correctly classified samples, while we obtained the same result using each of the seven gene pairs directly selected from the training set without the LOOCV procedure. Of note, six of the eight genes selected in this earlier study were also identified in the present study. The six genes are PTGIS, CD200, TACSTD1, TTF1, ANXA8, and CALB2, the first three of which have a 100% depended degree.

The genes selected by our method are associated primarily with the pathogenesis of MPM or ADCA or some other tumor. According to our rules, JUP, CD24, PRKCD, MAPK13, TACSTD2, DKFZP564O0823 protein, TACSTD1, CEACAM1, XBP1, TTF1, SFTPB, AGR2, ELF3, EVI1, and CDA are highly expressed in ADCA, while EGF, SPTAN1, FLNC, PTGIS, FBXL7, CD200, AP2 M1, ANXA8, HAS1, CALB2, GFPT2, KIAA0427, C1S, EIF4G3, TGM1, Adamts3, hypothetical protein dJ465N24.2.1, and AP3S1 are highly expressed in mesothelioma. CALB2 encodes calretinin, which is a component of several immunohistochemical panels currently used in the diagnosis of MPM and lung cancer [15]. HAS1 is a member of gene family HA, which has been correlated with tumor metastasis. In [51], HAS1 was identified as a prognostic gene for mesothelioma. In [52], HAS1 belongs to the list of the genes with elevated expression levels in C1 MPM tumors. We have one rule arising from HAS1: if g(HAS1) > 7.3, then MPM. This rule is consistent with the results of [51,52]. ANXA8, PTGIS, and CLAB2 are also marked as more highly expressed genes in C1 MPM tumors [52]. These observations are supported by the following rules of the present study: if g(ANXA8) > 130.8, then MPM; if g(CALB2) > 490.5, then MPM; if g(PTGIS) > 193.25, then MPM. Other genes that we chose (e.g., CD24, TACSTD1, TACSTD2, CEACAM1, and PRKCD) are correlated with lung carcinoma or other tumors. TTF1 is a transcription factor that regulates the expression of multiple genes involved in lung development. It is preferentially expressed in ADCAs of the lung and has been investigated as a potential prognostic parameter in patients with lung cancer [53-56].

#### Prostate Cancer dataset

Regarding the Prostate Cancer dataset, a previous study reported a 95% prediction rate using a gene pair [14]. The best classification results on the dataset, based on three different decision tree approaches (Single C4.5, Bagging C4.5, and AdaBoost C4.5), are 67.65%, 73.53%, and 67.65%, respectively [11]. In [13], a 97% classification result was reported, but the employed gene numbers were not provided. In [18], the authors built predictors using a *k*-NN algorithm, and achieved 77% and 86% prediction accuracy on the test set with 4 and 16 genes, respectively. Table 12 summarizes the best results of classification on the dataset.

**Table 12:** Comparison of best classification accuracy for the Prostate Cancer dataset

**Methods (feature selection + classification)**	**#Selected genes**	**#Correctly classified samples (accuracy)**	**Rule-based classifier**
depended degree + decision rules [this work]	1	31 (91.18%)	yes
		
	2	27 (79.41%)	

TSP [14]	2	32 (94.12%)	yes

PCLs [50]	unknown	33 (97.06%)	yes

discretization + Single C4.5 [11]	unknown	23 (67.65%)	yes

discretization + Bagging C4.5 [11]	unknown	25 (73.53%)	yes

discretization + AdaBoost C4.5 [11]	unknown	23 (67.65%)	yes

RCBT [13]	unknown	33 (97.06%)	yes

SVMs [13]	unknown	27 (79.41%)	no

signal to noise ratios + *k*-NNs [18]^d^	4	26 (77.2%)	no
		
	16	29 (85.7%)	no

^d^In [18], as both raw and normalized datasets were used, two groups of prediction results were obtained. Here, we chose their results from the normalized dataset. Another small difference is that we obtained the dataset from the Kent Ridge Bio-medical Data Set Repository, where the prostate test set includes 25 tumor and 9 normal samples instead of the 27 tumor and 8 normal samples studied in [69]. To facilitate comparison, the correctly classified sample numbers were calculated according to the total of 34 samples.

In the Prostate Cancer dataset, we identified 13 genes using the LOOCV approach. Seven of the eight single genes had relatively good classification performance, of which five genes had established names: NRP2, TMSB15A, PEDF, FAM107A and TGFB3. Our rules imply that TMSB15A, also named thymosin beta15, is highly expressed, while NRP2, PEDF, FAM107A and TGFB3 are expressed at low levels in tumor tissue. As revealed in [57], thymosin beta15 levels are elevated in human prostate cancer and correlate positively with the Gleason tumor grade. Thymosin beta 15 may represent a potential new biochemical marker for the progression of human prostate cancer; our rules strengthen this perspective. Previous investigations have revealed that PEDF expression is negatively correlated with tumor malignancy [58-62]; our rules support this viewpoint. FAM107A has been consistently reported to be downregulated in human cancer [63,64]; that conforms to our rules. In the gene pairs, our rules indicate that KIAA0762 is downregulated, while TUBB and RGS10 are upregulated in tumor tissue; however, there exists insufficient evidence to directly link the three genes with prostate cancer.

The three genes that we identified directly from the training set are hepsin (37639_at), KIAA0977 (41755_at), and S100A4 (38087_s_at). Hepsin performs reasonably well in differentiating two classes of samples, and the latter two genes are good indicators of normal samples. Hepsin is the human hepatoma mRNA for serine protease. Numerous studies have revealed that it is closely linked to prostate cancer. Hepsin is widely reported to be highly over-expressed in more than 90% of human prostate tumors, making it a significant marker and a target for prostate cancer [65-72]. In [18], hepsin was identified as the first over-expressed gene in tumor samples and was selected as one of 16 genes used for creating a prediction model. All of these outcomes strongly support our rules involved in hepsin. Another gene, KIAA0977, has also been listed as a highly expressed gene in tumor samples [18]. The third gene, S100A4, was associated with cancer pathogenesis, chromosomal rearrangements and altered expression of which have been implicated in tumor metastasis [73-75]. In [18], S100A4 was identified as one of the highly expressed genes in normal samples and chosen as one member of a 16-gene model of prediction. In addition, [76] noted that S100A4 protein was not expressed in benign or malignant prostatic epithelium or in LNCaP and Du145 cells. Our rules related to this gene support these previous findings. A surprising result is that many observations have revealed that S100A4 is over-expressed in most other tumors [77-82], yet in [76] the authors suggested that the mechanism of changes in the expression level of S100A4 may involve methylation of the S100A4 gene.

#### Breast Cancer dataset

In the Breast Cancer dataset, our best LOOCV accuracy was 74.34%, and the highest classification accuracy in the test set was 84.21% with one gene. In [19], the authors reported 83.33% LOOCV accuracy and 89.47% accuracy in the test set using the 70-gene predictor. These prediction results are moderately superior to those attained in the present study, although using a much larger number of genes. Likewise, Tan et al. [11] obtained a slightly better classification outcome than that of the present study, although they used far more genes. Table 13 lists some of the best classification results for this dataset, as obtained using a variety of methods.

**Table 13:** Comparison of best classification accuracy for the Breast Cancer dataset

**Methods (feature selection + classification)**	**#Selected genes**	**#Correctly classified samples (accuracy)**	**Rule-based classifier**
*α *depended degree + decision rules [this work]	1	16 (84.21%)	yes

TSP [14]	2	79.38%^e^	yes

RBF [50]	67	79.38%^e^	yes

discretization + decision trees [11]	unknown	17 (89.47%)	yes

correlation coefficient [19]	70	17 (89.47%)	no

^e^LOOCV result in the total of 97 samples.

In this dataset, we identified eight genes with relatively high individual classification performance. Our rules indicated that the overexpression of ATP5G3, TSPYL5, or NPC1 means an unfavorable prognosis, while the overexpression of HS1119D91, Contig38726_RC, PIB5PA, Contig51517_RC, or LOC63923 implies a favorable prognosis. TSPYL5 had the best classification accuracy in our model; it was also chosen as one of 70 prognostic marker genes and ranked first according to the correlation coefficient with the two prognostic groups [19]. It follows that our gene selection approach is reasonable. In [83], the authors proposed a prognostic predictor of breast cancer with multiple fuzzy neural models using the same dataset. Surprisingly, although these methods are distinct from those of the present study, there is an overlap of 3 genes between the 10 highest-ranked genes they chose for prediction and our 8-gene group.

#### Leukemia dataset 2

Although this dataset is involved in a multi-class classification problem, we still achieved relatively good classification outcomes. Our best prediction rate was 93.33% in the test set and 89.47% LOOCV accuracy in the training set, each by one gene, compared with a 90% prediction rate in the test set by 100 genes and 95% LOOCV accuracy in the training set by 40 genes, as reported by Armstrong et al. [84]. In addition, Wang et al. reported 100% LOOCV accuracy in all 72 samples using 26 genes; however, their methods were not verified by an independent test set. These outcomes are presented in Table 14.

**Table 14:** Comparison of best classification accuracy for the Leukemia dataset 2

**Methods (feature selection + classification)**	**#Selected genes**	**#Correctly classified samples (accuracy)**	**Rule-based classifier**
*α *depended degree + decision rules [this work]	1	14 (93.33%)	yes

HykGene + *k*-NNs, SVMs, C4.5, NB [85]	26	100%^f^	no^i^

signal to noise ratios + *k*-NNs [20]	40	95%^g^	no
		
	100	9 (90%)^h^	

^f^LOOCV result in a total of 72 samples.

^g^LOOCV result in a total of 57 training samples.

^h^In [20], only 3 of 8 AML testing samples in the dataset were mentioned. Thus, their test set contained 10 rather than 15 samples.

^i^Except for C4.5, all the others are not rule-based classifiers.

Regarding the Leukemia dataset 2, each chosen gene induced 3 rules with the following form: if g(x) > a, then class 1; if b < g(x) ≤ a, then class 2; if g(x) ≤ b, then class 3. That is, if the expression level of gene x in a sample is relatively high, then the sample is assigned to class 1; if the expression level is moderate, then the sample is assigned to class 2; if the expression level is relatively low, then the sample is assigned to class 3. According to the standard, we predicted the class of every sample based on its expression value on the chosen genes. In total, we identified 21 genes with comparatively strong prediction power. Of these genes, 36239_at (OBF-1) and 31886_at (human placental cDNA coding for 5' nucleotidase) are also contained in the best 26-gene prediction model proposed in [85]. It is noteworthy that OBF-1 was ranked as the top of these 26 genes, and it yields the best prediction outcome in our methods. This finding demonstrates that our decision-rule-based classification approach is superior to the clustering analysis-based classification approach of [83], as we achieved a similar level of classification performance using just a single gene instead of 26. In addition, six of the genes identified using the present methods are mentioned as high-class discrimination genes in [20]. These six genes are OBF-1, CD24, MLCK, KIAA0867, SMARCA4, and cDNA wg66 h09. Indeed, our rules induced by each of the six genes are well in accordance with the outcomes presented in [20], demonstrating that these genes are highly expressed in ALL, moderately expressed in MLL, and expressed at a low level in AML.

In summary, we have identified some important genes that not only possess potent classification ability but also are closely associated with the pathogenesis of specific or general cancers in every dataset. In the Leukemia dataset 1, significant genes such as Zyxin and MGST1, frequently identified by previous researchers, were also identified in the present study. At the same time, we selected some genes rarely identified by other methods (e.g., TCRA, KIAA0159, and MB-1), which have been proven to correlate directly or indirectly with AML-ALL class prediction. Our results demonstrate that the genes with excellent performance in AML-ALL classification are not only the markers of hematopoietic lineage, but also related to general cancer pathogenesis. Therefore, the genes we have identified, which are useful for AML-ALL classification, are also indicators of cancer pathogenesis and pharmacology. This is consistent with the conclusion of Golub et al. [2]. In the Lung Cancer dataset, we succeeded in identifying highly discriminative genes (e.g., CALB2, HAS1, and ANXA8) implicated in the pathogenesis of MPM, ADCA, or other tumors. In the Prostate Cancer dataset, we identified some important genes with significant biological relevance, such as TMSB15A, PEDF, hepsin, KIAA0977, and S100A4. In particular, hepsin, which has the highest depended degree, has been reported to have significant involvement in the pathogenesis of prostate cancer. In the Breast Cancer dataset, TSPYL5 was regarded as the most valuable prognostic marker by our methods and by the correlation-based approach used in [19]. In the Leukemia dataset 2, we identified OBF-1 and others, which excellently separate ALL, MLL, and AML. Overall, the majority of genes relevant to tumors encode proteins functioning in cell growth, motility and differentiation, apoptosis, angiogenesis, metabolism, chromosomal rearrangement and translocation, and immune reactions.

## Discussion

Microarray-based cancerous gene classification is a particular classification problem: the quantity of features (genes) greatly exceeds the number of instances (samples). As the majority of features are redundant for the classification task, feature selection is of vital importance. At the same time, the discovery of important gene markers relevant to cancer remains a significant task. To this end, we proposed a method of feature selection based on the depended degree of attributes by classes, by which we screened single or double informative genes for classification. We built classifiers on the basis of the decision rules arising from these genes or gene pairs. Using just a small number of features, we gained high-quality solutions to classification problems in the analysis of high-dimensional gene expression data.

In general, our approach has advantages over other methods. For example, our methods are based on rules. In contrast to non-rule-based methods (e.g., SVMs, ANNs, GAs, *k-*NNs and NB) rule-based methods are understandable and logical, so that biologists and clinicians are more inclined to adopt them. More importantly, as we utilize very few genes (one or two) to construct classification rules, the derived classifiers are quite simple and easily understood. Hence, our rule-based method has an advantage over other rule-based methods that involve more complicated rules.

Our work is consistent with the opinion expressed in [86,87]: simple approaches perform well in microarray-based cancer prediction. This opinion is supportive of the principle of Occam's razor. It is not strange that single or double genes can result in accurate classification of cancer, as the single genes or gene pairs might be the potential biomarkers of cancer [17]. In contrast, when complex prediction models achieve highly accurate prediction rates using a large number of genes, it is difficult to assess which genes are the significant biomarkers of cancer. In fact, molecular classification of cancer is a specific classification problem, as it incorporates essential double implications: classification and identifying biomarkers of cancer. Although accurate classification must be guaranteed, the detection of biomarkers is also important, sometimes even more so than accuracy; otherwise, the (accurate) classification results have only limited significance. Because simple classification models may be advantageous in finding important biomarkers with a high classification accuracy, it is worthwhile applying simple prediction approaches rather than complex methods for the molecular classification of cancer. Furthermore, it is better to utilize simple rule-based classification methods because of their interpretability.

It should be noted that because we only verified the classification accuracy using one independent test set for every dataset, the stability of the classifier was not assessed. That is, if the different training and test sets are chosen, the classification results maybe vary, although not necessarily significantly deviate from our estimates. Therefore, the present classification accuracies only roughly reflect the quality of our classifiers. One more unbiased estimate should be based on the average of the results obtained by repeating the partition of samples between training and test set many times, which is time consuming for our methods.

## Conclusion

Our microarray-based cancer classification methods are simple and interpretable relative to most other approaches, since our classifiers are based on decision rules, and the decision rules are based on single or double genes. We demonstrated the efficacy of our methods by their application to several well-known gene expression datasets. In these datasets, our methods identified the single genes or gene pairs that perform well in distinguishing different classes of cancer. Moreover, a large proportion of the genes screened by our methods may have biological relevance to malignancy or cell type, meaning that they can be regarded as candidate biomarkers of cancer.

Generally speaking, simple classification models are capable of giving good performance in most classification problems, including the molecular classification of cancer, if a small number of features are correctly selected [6,12,14,88,89]. The present results lend support to this notion. One recommended follow-up study is to combine our methods with other established machine-learning algorithms to address the problem of molecular classification of cancer.

## Abbreviations

LOOCV: Leave-One-Out Cross-Validation; CNS: Central Nervous System; SVMs: Support Vector Machines; DA: Discriminant Analysis; ANNs: Artificial Neural Networks; GAs: Genetic Algorithms; NB: Naive Bayes; *k*-NNs: *k*-Nearest Neighbors; EPs: Emerging Patterns; AML: Acute Myeloid Leukemia; ALL: Acute Lymphoblastic Leukemia; MLL: Mixed-Lineage Leukemia; MPM: Malignant Pleural Mesothelioma; ADCA: adenocarcinoma; MDL: Minimum Description Length; PNNs: Probabilistic Neural Networks; CBF: Correlation-Based Feature; MAMA: MAximal MArgin linear programming; RCBT: Refined Classification method Based on Top-*k *covering rule groups; PLS: Partial Least Squares; LD: Logistic Discrimination; QDA: Quadratic Discriminant Analysis; PCLs: Prediction by Collective Likelihoods.

## Competing interests

The authors declare that they have no competing interests.

## Authors' contributions

XW designed and performed research. XW wrote programming codes and analyzed data. XW wrote the paper. OG first proposed the idea of using the AML-ALL dataset to realize our algorithm. OG helped to draft the manuscript. OG provided helpful instructions in programming and wrote partial codes. Both authors read and approved the final manuscript.

## Pre-publication history

The pre-publication history for this paper can be accessed here:



## Supplementary Material

Additional file 1 **The rules derived from each of the 12 gene pairs identified in the Leukemia dataset 1.** Click here for file

Additional file 2 **The top 87 genes with depended degrees of no less than 0.5 in the training set of the Leukemia dataset 1.** Click here for file

Additional file 3 **The rules produced by each of the 16 genes and 25 gene pairs identified in the Lung Cancer dataset.** Click here for file

Additional file 4 **The experimental results and the seven gene pairs with high classification accuracy in the test set of the Lung Cancer dataset, identified without LOOCV.** Click here for file

Additional file 5 **The classification rules generated by each of the eight genes and three gene pairs identified in the Prostate Cancer dataset.** Click here for file

Additional file 6 **The top 20 genes ranked based on depended degree in the training set of the Prostate Cancer dataset.** Click here for file

Additional file 7 **The classification rules generated by each of the eight genes identified in the Breast Cancer dataset.** Click here for file

Additional file 8 **The classification rules generated by each of the 21 genes identified in the Leukemia dataset 2.** Click here for file
