# Supplementary material for: Accurate molecular classification of cancer using simple rules
Source: BMC Med Genomics. 2009 Oct 30;2:64. doi: 10.1186/1755-8794-2-64 (PMC2777919; doi:10.1186/1755-8794-2-64)
Supplement: Additional file 4 — The experimental results and the seven gene pairs with high classification accuracy in the test set of the Lung Cancer dataset, identified without LOOCV. [file 1755-8794-2-64-S4.doc]

**Table S1 - The experimental results in the Lung Cancer dataset**

| Threshold | #Genes | #Gene pairs | #Result gene pairs |
| --- | --- | --- | --- |
| 13 | 61 vs.77 | 743 vs.1839 | 4 vs.4 |
| 12 | 126 vs.142 | 2603 vs.4739 | 4 vs.4 |
| 11 | 188 vs.204 | 4787 vs.7915 | 4 vs.4 |
| 10 | 296 vs.312 | 8988 vs.13844 | 4 vs.4 |
| 9 | 447 vs.463 | 14055 vs.21327 | 5 vs.5 |
| 8 | 649 vs.665 | 19574 vs.30078 | 7 vs.7 |
| 7 | 923 vs.939 | 25347 vs.40235 | 7 vs.7 |
| 0 | 1428 vs.1444 | 33390 vs.56358 | 7 vs.7 |

Two contrast groups: excluding the 16 genes with 100% depended degree vs. including them.

The size of the positive region induced by each chosen gene must be greater than the threshold.

#Genes: the number of the chosen genes according to the threshold.

#Gene pairs: the number of the gene pairs with 100% depended degree in the training set.

#Result gene pairs: the number of the gene pairs classifying the test set with at most one error.

**Table S2 - Seven pairs of genes with high classification accuracy in the test set of the Lung Cancer dataset identified without LOOCV**

| 1st – 2nd Probe ID | #Correctly-classified samples | Classification accuracy (%) |
| --- | --- | --- |
| 291_s_at - 37954_at | 148 | 99 |
| 32424_at - 37157_at | 148 | 99 |
| 32424_at - 39640_at | 148 | 99 |
| 32424_at - 39755_at | 148 | 99 |
| 36685_at - 37157_at | 148 | 99 |
| 36685_at - 39640_at | 148 | 99 |
| 37157_at - 39640_at | 148 | 99 |
